# Interleukin-9 Facilitates Osteoclastogenesis in Rheumatoid Arthritis

**DOI:** 10.3390/ijms221910397

**Published:** 2021-09-27

**Authors:** Santanu Kar, Ranjan Gupta, Rajesh Malhotra, Vijay Sharma, Kamran Farooque, Vijay Kumar, Sushmita Chakraborty, Dipendra Kumar Mitra

**Affiliations:** 1 Department of Orthopaedics, All India Institute of Medical Sciences, New Delhi 110029, India; karsantanu109@gmail.com (S.K.); rmalhotra62@hotmail.com (R.M.); drvijaysharmatrauma@gmail.com (V.S.); kamran.farooque@gmail.com (K.F.); vijayaiims@yahoo.com (V.K.); 2 Department of Rheumatology, All India Institute of Medical Sciences, New Delhi 110029, India; dr.guptaranjan@gmail.com; 3 Department of Transplant Immunology and Immunogenetics, All India Institute of Medical Sciences, New Delhi 110029, India

**Keywords:** rheumatoid arthritis, interleukin-9, osteoclast, matrix metalloproteinases, osteoclastogenesis, differential gene expression

## Abstract

In rheumatoid arthritis (RA), inflammatory cytokines play a pivotal role in triggering abnormal osteoclastogenesis leading to articular destruction. Recent studies have demonstrated enhanced levels of interleukin-9 (IL-9) in the serum and synovial fluid of patients with RA. In RA, strong correlation has been observed between tissue inflammation and IL-9 expression in synovial tissue. Therefore, we investigated whether IL-9 influences osteoclastogenesis in patients with RA. We conducted the study in active RA patients. For inducing osteoclast differentiation, mononuclear cells were stimulated with soluble receptor activator of NF-kB ligand (sRANKL) and macrophage-colony-stimulating factor (M-CSF) in the presence or absence of recombinant (r) IL-9. IL-9 stimulation significantly enhanced M-CSF/sRANKL-mediated osteoclast formation and function. Transcriptome analysis revealed differential gene expression induced with IL-9 stimulation in the process of osteoclast differentiation. IL-9 mainly modulates the expression of genes, which are involved in the metabolic pathway. Moreover, we observed that IL-9 modulates the expression of matrix metalloproteinases (MMPs), which are critical players in bone degradation. Our results indicate that IL-9 has the potential to influence the structural damage in the RA by promoting osteoclastogenesis and modulating the expression of MMPs. Thus, blocking IL-9 pathways might be an attractive immunotherapeutic target for preventing bone degradation in RA.

## 1. Introduction

Rheumatoid arthritis (RA) is an inflammatory bone disease characterized by uncontrolled synovial inflammation resulting in subsequent destruction of bone and cartilage [1]. Patients with RA frequently experience poor skeletal health, increased risk of fractures, and osteoporosis due to the imbalance in bone remodeling, which is an important phenomenon for maintaining bone integrity [2,3]. Bone remodeling is a complex and dynamic process. This process is tightly controlled by two types of bone cells, osteoclasts that mediate bone resorption, and osteoblasts, which participate in the formation of new bone matrix. Under physiological conditions, complex interplay of cytokines modulates the formation and function of these bone cells in order to maintain the skeletal homeostasis. But in RA, exaggerated inflammatory cytokines disrupt the bone homeostasis due to enhanced osteoclast formation and function, resulting in more bone decay over bone formation [4,5].

Osteoclasts are multinucleated cells derived from myeloid precursor cells. Formation of osteoclasts from their precursor cells is predominantly controlled by two cytokines—macrophage colony stimulating factor (M-CSF) and receptor activator of NF-kB ligand (RANKL) [6,7]. After attaching to the bone matrix, osteoclasts mediate bone degradation by secreting proteinases and by creating an acidic milieu.

Several pieces of evidence derived from the pathological samples of RA patients and animal models of RA support the critical role of osteoclasts in the pathogenesis of RA [8,9,10,11]. In RA, the involvement of osteoclasts in bone erosion is also evident from studies using mice deficient in factors essential for osteoclast formation. These osteoclast-lacking mice when induced for arthritis using serum transfer, showed protection from bone degradation despite having inflammation [12]. In addition, osteoclast-deficient mice showed protection from bone erosion when crossed with tumor necrosis factor-α (TNF-α)-expressing transgenic mice, which spontaneously develop arthritis [13]. These observations clearly indicate the importance of osteoclasts in inflammatory bone diseases.

The process of osteoclastogenesis is influenced by various local and systemic factors. In RA, prominent proinflammatory cytokines such TNF-α, interleukin (IL)-1, IL-6, and IL-17 have been implicated as enhancing the process of osteoclastogenesis [14,15,16,17,18,19]. Thus, in the inflamed joints of RA patients, accumulated inflammatory cytokines due to the exaggerated immune activation contribute to the enhanced osteoclast formation.

Recently, an increased level of IL-9 has been observed at the disease site as well as in the circulation of patients with RA [20,21,22]. This cytokine is a member of the common γ-chain (γc) family [23]. It mediates pleiotropic function by binding to a heterodimeric receptor, composed of an IL-9 receptor alpha (IL-9Rα) chain and a gamma chain (γC). The cytokine is profusely secreted by a new subset of T-helper (Th) cells know as Th-9 cells, but is also produced by Th-17 cells, important players in mediating inflammation and osteoclastogenesis in RA. In RA, the frequencies of both Th-9 and Th-17 cells strongly correlate with the disease activity [21,24]. Additionally, IL-9 was observed to prolong the survival of neutrophils and augment the functions of inflammatory T cells in RA [21]. From these observations, it is conceivable that IL-9 plays an important role in the disease pathogenesis. However, the effect of IL-9 on osteoclastogenesis in RA remains to be determined.

In this study, we investigated the impact of IL-9 on MCSF and RANKL-mediated osteoclastogenesis in RA. Most studies with human samples are performed with cells derived from peripheral blood (PB), but they do not provide a clear reflection of the disease pathology. Therefore, we analyzed the osteoclastogenic potential of IL-9 on cells derived from both peripheral blood (PB) and synovial fluid (SF) of patients with RA. Additionally, we performed transcriptome analysis to understand the differential gene expression induced by IL-9 in order to influence osteoclastogenesis.

Here, we demonstrate that IL-9 promotes MCSF/RANKL-mediated osteoclastogenesis in RA by enhancing the production of TNF-α and by modulating the expression of genes involved in the metabolic pathways.

## 2. Results

### 2.1. Patient Characteristics

The patients included in the study were examined in the Out-Patient Department (OPD) and the details of the clinical profile have been summarized in Table 1. The mean age of the patients was 45.06 ± 8.74 years with 5 males and 10 females. The average duration of the disease symptoms was 9.76 ± 3.88 years. Mean disease activity score (DAS) 28 score was 5.21 ± 0.89. The disease activity at first presentation was determined using ESR and CRP. Mean ESR was 31.73 ± 14.6 and mean CRP was 16.14 ± 15.1; suggesting higher disease activity in the patients. All the patients received disease-modifying anti-rheumatoid agents or biological-like anti-TNFα drugs or combination therapy. Radiological joint erosions were present in all the patients except two cases. Age- and sex-matched healthy subjects (5 males and 10 females) were recruited for the study and their mean age was 41.2 ± 8.04 years.

### 2.2. Influence of IL-9 on M-CSF- and RANKL-Induced Osteoclastogenesis

**Figure 1:**
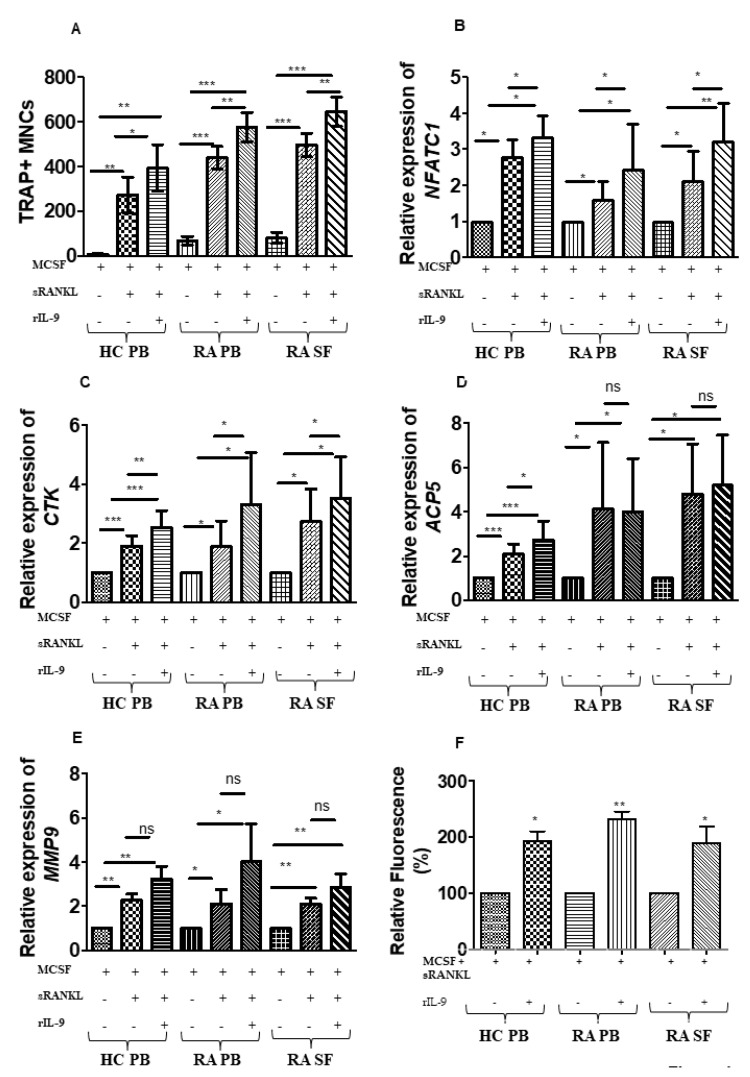
Interleukin (IL)-9 enhances osteoclast formation and function in cells derived from RA and HC. (**A**) Cells derived from peripheral blood (PB) of healthy control (HC); cells derived from PB and synovial fluid (SF) of patients with RA were treated as indicated with macrophage colony-stimulating factor (M-CSF; 25 ng/mL), soluble receptor activator of nuclear factor κB ligand (sRANKL; 50 ng/mL), or IL-9 (100 ng/mL) for 21 days. Half of the culture medium was replenished with fresh culture medium containing stimulating factors (M-CSF, sRANKL, and rIL-9) at 4-day intervals. Cells were then fixed and stained for tartrate-resistant acid phosphatase (TRAP). Using a light microscope, multinucleated (≥3 nuclei) TRAP+ cells were counted manually. Graph shows TRAP+ multinucleated cells (MNCs) (mean  ±  SD; n  =  5). Statistical analysis was performed using paired Student’s *t*-test comparing M-CSF/sRANKL/rIL-9-treated cells to M-CSF/sRANKL, M-CSF, or M-CSF/sRANKL-treated cells to M-CSF (*: *p*  ≤  0.05; **: *p*  ≤  0.005; ***: *p*  ≤  0.0005). (**B**–**E**) Cells derived from PB of HC, PB, and SF of patients with RA were stimulated as indicated with M-CSF (25 ng/mL), sRANKL (50 ng/mL), or IL-9 (100 ng/mL) for 3 days. Cells were then lysed followed by RNA extraction and cDNA preparation. Quantitative real-time PCR (RT-PCR) was performed for osteoclast-specific marker genes, nuclear factor of activated T-cells, cytoplasmic 1 (*NFATc1*), cathepsin K (*CTSK*), acid phosphatase 5, tartrate-resistant (*ACP5*), and matrix metalloproteinase-9 (*MMP-9*). The graphs represent the relative expression of *NFATc1*, *CTSK*, *ACP5,* and *MMP-9* (mean  ±  SD; n  =  8). Statistical analysis was performed using paired Student’s *t*-test comparing M-CSF/sRANKL/rIL-9-treated cells to M-CSF/sRANKL-, M-CSF-, or M-CSF/sRANKL-treated cells to M-CSF (*: *p*  ≤  0.05; **: *p*  ≤  0.005; ***: *p*  ≤  0.0005; ns –non significant). (**F**) Cells derived from PB of HC, PB, and SF of patients with RA were stimulated with M-CSF (25 ng/mL), sRANKL (100 ng/mL), or IL-9 (100 ng/mL) as indicated for 24 days. Half of the culture medium was replenished with fresh culture medium containing stimulating factors (M-CSF, sRANKL, and rIL-9) at 4-day intervals. Using a fluorometer, fluorescence intensity of the culture supernatant was measured. The graph represents the relative fluorescence intensity (mean  ±  SD; n  =  3). Statistical analysis was performed using paired Student’s *t*-test comparing M-CSF/sRANKL/rIL-9-treated cells to M-CSF/sRANKL-treated cells. (*: *p*  ≤  0.05; **: *p*  ≤  0.005).

In RA, IL-9 influences the pathogenesis of the disease by enhancing the survival of neutrophils and by augmenting the inflammatory cytokine producing T cells (21). Here, we investigated the effect of IL-9 on osteoclastogenesis in cells derived from both peripheral blood (PB) and synovial fluid (SF) of patients with RA. As a control, we also checked the effect of IL-9 on osteoclastogenesis in cells derived from PB of healthy controls (HC). In vitro differentiations of human monocytes into osteoclasts require stimulation with soluble (s) RANKL and M-CSF. Thus, cells were cultured for 21 days with M-CSF alone (M-CSF) or M-CSF along with sRANKL (M-CSF + sRANKL) or in a combination of M-CSF, sRANKL and recombinant (r) IL-9 (M-CSF + sRANKL + rIL-9). Tartrate-resistant acid phosphatase (TRAP)-positive cells with three or more nuclei were scored as osteoclasts. We observed a significant increase in the number of osteoclasts with rIL-9 stimulation along with M-CSF and sRANKL as compared to the stimulation with M-CSF and sRANKL in PB of HC, PB, and SF of patients with RA (Figure 1A; Supplementary Figure S1). We next checked the expression of osteoclast markers in the cells treated with M-CSF and sRANKL in the presence or absence of rIL-9 (Figure 1B–E). Real-time PCR analysis revealed that rIL-9 stimulation along with M-CSF and sRANKL significantly enhanced the expression of nuclear factor of activated T cells, cytoplasmic 1 (NFATc1), a master transcription regulator of osteoclast differentiation in cells derived from PB of HC, PB, and SF of patients with RA (Figure 1B). Treatment with rIL-9 along with M-CSF and sRANKL also enhanced the expression of cathepsin K (CTSK), a potent protease in mediating bone resorption as compared to M-CSF/sRANKL-stimulated cells (Figure 1C). However, the expression of acid phosphatase 5, tartrate-resistant (ACP5), and matrix metalloproteinase (MMP)-9 was comparable in cells treated with M-CSF and sRANKL in the presence or absence of IL-9 in cells derived from PB of HC, PB, and SF of RA patients (Figure 1D and E). Next, we tested whether IL-9 stimulation along with M-CSF and sRANKL enhances the bone resorptive capacity of osteoclasts (Figure 1F). Using a fluorescent bone resorption assay, we checked the resorptive capacity of osteoclasts on fluoresceinated calcium-phosphate-coated plates by measuring the fluorescence intensity of the culture supernatant. Indeed, IL-9 stimulation along with M-CSF and sRANKL significantly enhanced the bone resorptive capacity of osteoclasts. The effect of IL-9 on osteoclastogenesis was comparable in cells derived from either circulation (PB) or inflammatory site (SF) of RA patients. Together, these observations suggest that IL-9 has the potential to enhance the RANKL-mediated differentiation of monocytes into functional osteoclasts.

### 2.3. IL-9 Enhances TNF-α Production during Osteoclastogenesis

**Figure 2:**
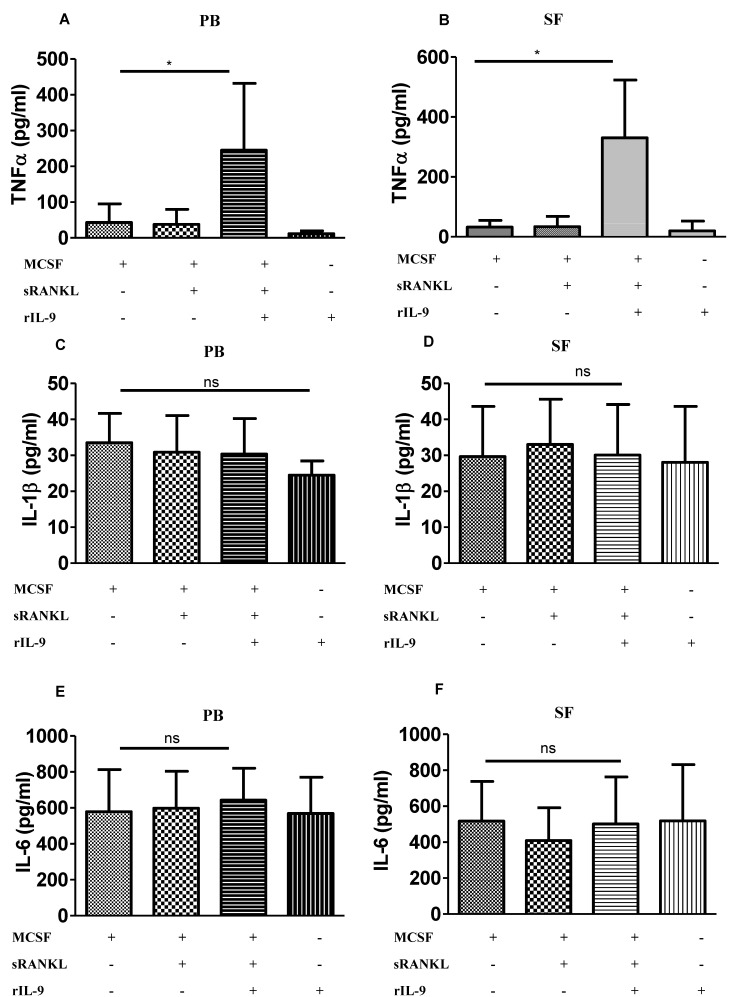
Interleukin (IL)-9 enhances TNF-α production during osteoclastogenesis in RA. (**A**–**F**) Cells derived from peripheral blood (PB) and synovial fluid (SF) of patients with RA were treated as indicated with macrophage colony-stimulating factor (M-CSF; 25 ng/mL), soluble receptor activator of nuclear factor κB ligand (sRANKL; 50 ng/mL), or IL-9 (100 ng/mL) for 4 days. Cell culture supernatants were analyzed for TNF-α (**A**,**B**), IL-1β (**C**,**D**), and IL-6 (**E**,**F**) using ELISA (mean  ±  SD; n = 8). Statistical analysis was performed using paired Student’s *t*-test comparing M-CSF/sRANKL/rIL-9-treated cells to M-CSF/sRANKL- or M-CSF-treated cells (*: *p*  ≤  0.05; ns–non significant).

In RA, IL-9 has been observed to enhance the frequency of TNF- α-producing T cells [21]. TNF-α is a potent osteoclastogenic cytokine as it not only enhances the RANKL-induced osteoclastogenesis but can mediate the process of osteoclastogenesis in a RANKL-independent manner [14]. In addition, TNF-α-expressing transgenic mice can spontaneously develop arthritis [25]. Therefore, we investigated whether IL-9 can influence the production of TNF-α, a potent osteoclastogenic cytokine in monocytes. Cells derived from PB and SF of patients with RA were stimulated with M-CSF alone (M-CSF), a combination of M-CSF and sRANKL (MCSF + sRANKL), or a combination of M-CSF, sRANKL, and rIL-9 (M-CSF + sRANKL + rIL-9) for 4 days. Cell culture supernatants were analyzed for TNF-α (Figure 2A,B). We observed that IL-9 stimulation along with M-CSF and RANKL enhanced the production of TNF-α both in PB-derived cells and SF-derived cells as compared to stimulation with M-CSF or M-CSF along with sRANKL.

Similar to TNF-α, the proinflammatory cytokines IL-1β and IL-6 are potent stimulators of RANKL-mediated osteoclast differentiation and bone resorption [26]. Thus, we checked whether IL-9 has the capacity to modulate the production of IL-1β and IL-6 during osteoclastogenesis. Cell culture supernatants from stimulated cells were analyzed for IL-1β (Figure 2C,D) and IL-6 (Figure 2E,F). IL-9 stimulation along with MCSF and sRANKL did not show any effect on the production of IL-6 and IL-1β. Thus, IL-9 influences osteoclastogenesis by inducing TNF-α in RA.

### 2.4. IL-9 Induces Differential Gene Expression during Osteoclastogenesis in RA

**Figure 3:**
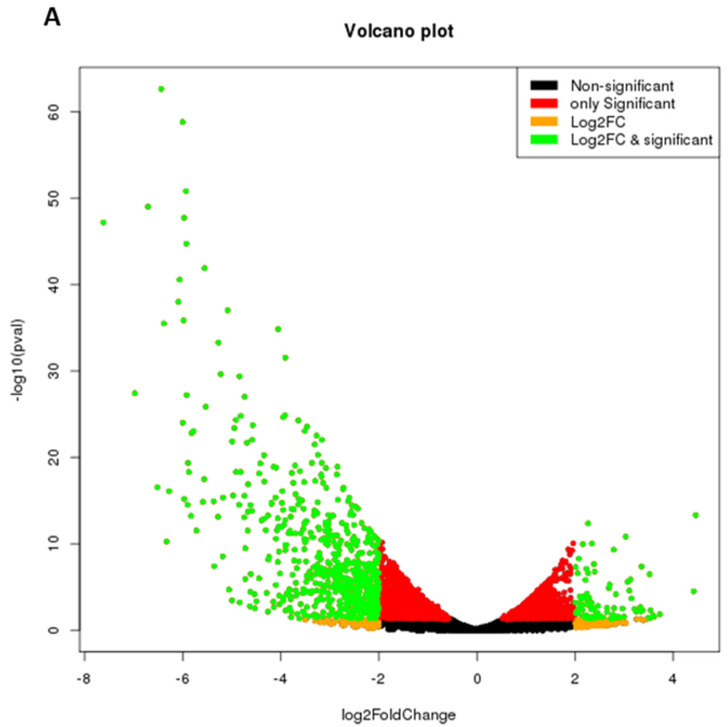

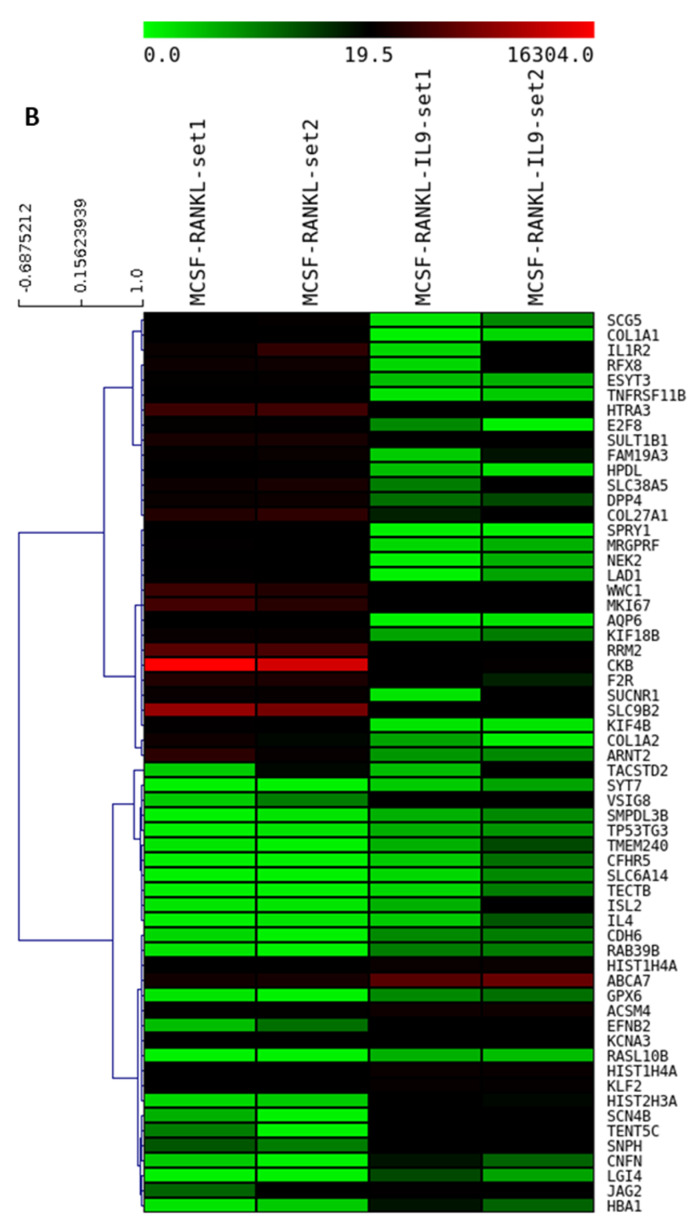
IL-9 mediated differential gene expression profile during osteoclastogenesis in RA. Cells derived from synovial fluid (SF) of patients with RA were treated with macrophage colony-stimulating factor (M-CSF; 25 ng/mL) and soluble receptor activator of nuclear factor κB ligand (sRANKL; 50 ng/mL) in the presence or absence of rIL-9 (100 ng/mL) for 4 days. After that RNA sequencing was performed with the treated cells as described in Materials and Methods. (**A**) Volcano plot of differentially expressed genes between M-CSF + sRANKL-treated cells and M-CSF + sRANKL + rIL-9-treated cells. (**B**) Heatmap of hierarchical cluster analysis of differentially expressed genes between M-CSF + sRANKL-treated cells and M-CSF + sRANKL + rIL-9-treated cells.

To check the effect of IL-9 on the differential gene expression profile during osteoclastogenesis in RA, cells derived from SF of patients with RA were stimulated with M-CSF along with sRANKL in the presence or absence of rIL-9. Differentially expressed genes (DEG) were identified between cells treated with M-CSF along with sRANKL in the presence or absence of rIL-9 using the DESeq software. For false discovery rate (FDR), the threshold level was defined as a less than 0.05. We observed 778 DEGs (genes filtered at 2 fold change and p value 0.05) between cells treated with M-CSF along with sRANKL in the presence or absence of rIL-9. Among them, 111 genes were upregulated and 667 genes were downregulated. Upregulated and downregulated DEGs are represented in volcano plots (Figure 3A). We also performed hierarchical cluster analysis; the upregulated and the downregulated genes are shown in the heatmap (Figure 3B). Analysis with the Kyoto Encyclopedia of Genes and Genomes (KEGG) pathway showed that IL-9-mediated differentially expressed genes were mostly involved in metabolic pathways (Supplementary Figure S2). These observations indicate that IL-9 influences osteoclast differentiation in RA by differentially regulating gene expression.

### 2.5. Validation of the IL-9-Induced Differentially Expressed Genes

**Figure 4:**
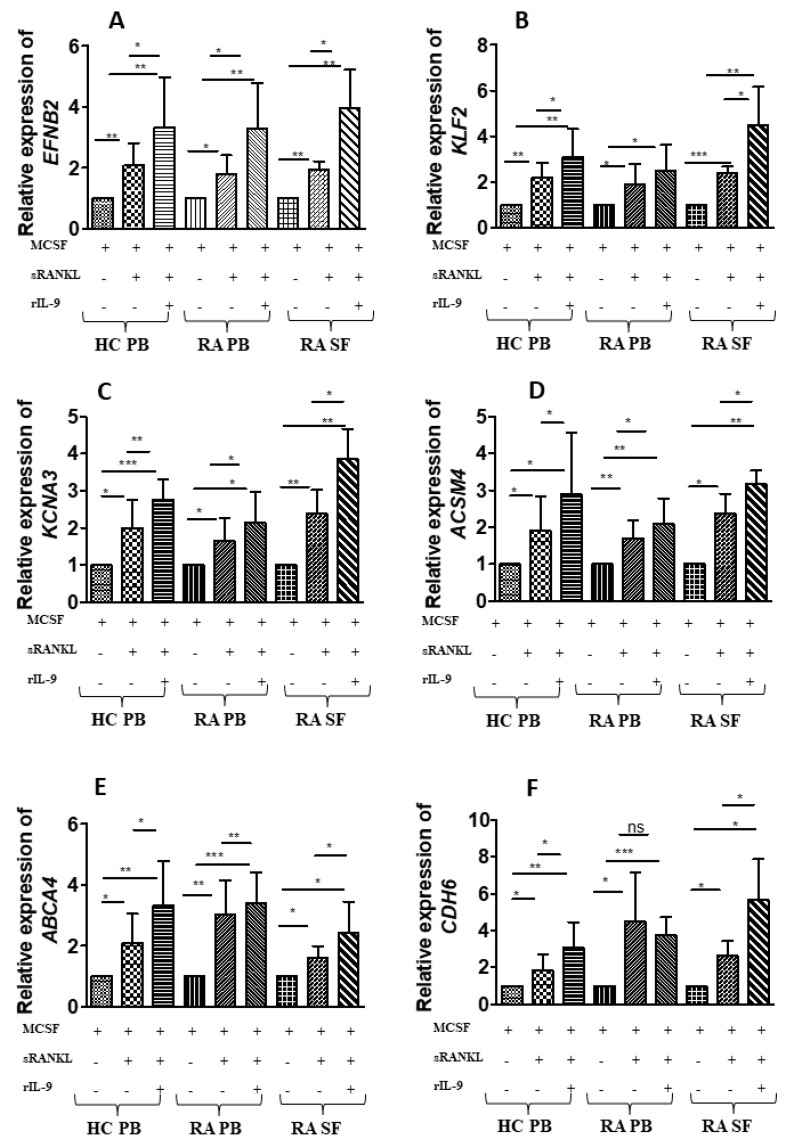
Validation that IL-9 induced six differentially expressed genes during osteoclast differentiation. (**A**–**F**) Cells derived from peripheral blood (PB) of healthy control (HC) and cells derived from PB and synovial fluid (SF) of patients with RA were treated as indicated with macrophage colony-stimulating factor (M-CSF; 25 ng/mL), soluble receptor activator of nuclear factor κB ligand (sRANKL; 50 ng/mL), or IL-9 (100 ng/mL) for 4 days. Cells were then lysed followed by RNA extraction and cDNA preparation from an equal quantity of RNA. Glyceraldehyde phosphate dehydrogenease (GAPDH) was used as an internal control for validation of the differentially expressed genes, as the expression of GAPDH showed least variation with stimulation. Quantitative real-time PCR (RT-PCR) was performed for ephrinB2 (EFNB2), ATP-binding cassette subfamily A member 7 (ABCA7), Krueppel-like factor 2 (KLF2), cadherin 6 (CDH6), acyl-coenzyme A synthetase (ACSM4), and potassium voltage-gated channel subfamily A member 3 (KCNA3). The graphs represent the relative expression of these genes (mean  ±  SD; n  =  8). Statistical analysis was performed using paired Student’s *t*-test comparing M-CSF/sRANKL/rIL-9-treated cells to M-CSF/sRANKL-, M-CSF-, and M-CSF/sRANKL-treated cells to M-CSF (*: *p*  ≤  0.05; **: *p*  ≤  0.005; ***: *p*  ≤  0.0005; ns–non significant).

Transcriptome analysis revealed differential gene expression between cell treated with M-CSF along with sRANKL in the presence or absence of rIL-9. From transcriptome analysis, we selected six upregulated genes with stimulation with M-CSF along with sRANKL in presence of rIL-9, which are relevant in the context of osteoclastogenesis and validated in more samples. PB-derived cells (HC and RA) and SF-derived cells (RA) were stimulated with either M-CSF alone or in combination with sRANKL in the presence and absence of rIL-9 for 4 days (Figure 4A–F). Treated cells were analyzed for the expression of ephrinB2 (EFNB2), ATP-binding cassette subfamily A member 7 (ABCA7), Krueppel-like factor 2 (KLF2), cadherin 6 (CDH6), acyl-coenzyme A synthetase (ACSM4), and potassium voltage-gated channel subfamily A member 3 (KCNA3) using real time PCR (Figure 4A–F). We validated that IL-9 enhances the expression of these genes in order to augment the M-CSF/RANKL-mediated osteoclastogenesis.

### 2.6. IL-9 Modulates the Expression of Matrix Metalloproteinases of Bone Cells

Matrix metalloproteinases (MMPs) are the critical player in the degradation of the organic matrix of the bone and play a fundamental role in the pathological destruction of joint tissues in RA. Several MMPs have been identified over recent years and are divided into four subgroups—collagenases, gelatinases, stromelysins, and membrane-type metalloproteinases. Therefore, we investigated the influence of IL-9 on the expression of selected MMPs (MMP-1, -2, -3, -8, -9, -12, -13, and -16), which are known to be involved in the pathogenesis of RA [27].

**Figure 5:**
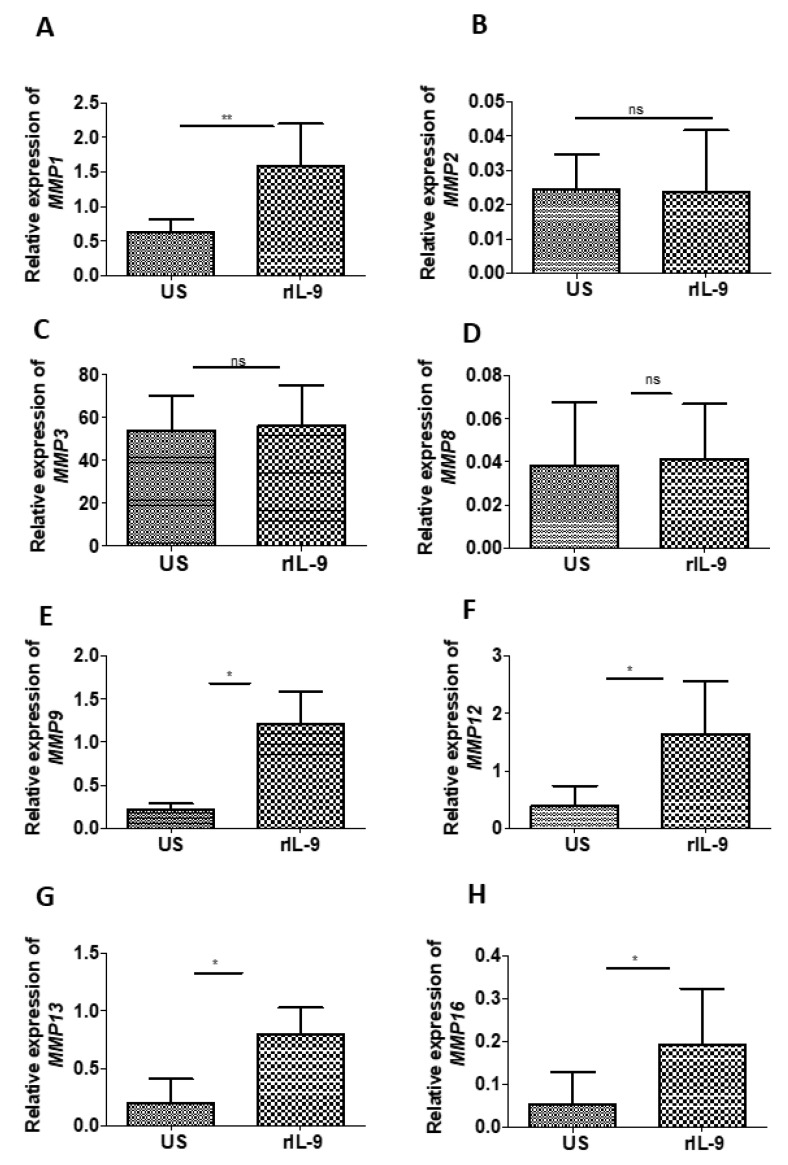
Effect of IL-9 on the expression of matrix metalloproteinases (MMPs). (**A**–**H**) Bone explant culture was stimulated with and without rIL-9 (100 ng/mL) for 5 days. Cells were then lysed and homogenized, followed by RNA extraction and cDNA preparation from an equal quantity of RNA. Glyceraldehyde phosphate dehydrogenease (GAPDH) was used as an internal control as the expression of GAPDH showed the least variation with stimulation. Quantitative real-time PCR (RT-PCR) was performed for MMP-1 (**A**), MMP-2 (**B**), MMP-3 (**C**), MMP-8 (**D**), MMP-9 (**E**), MMP-12 (**F**), MMP-13 (**G**), and MMP-16 (**H**). The graphs represent the relative expression of these genes (mean  ±  SD; n  =  6). Statistical analysis was performed using paired Student’s *t*-test comparing treated cells to untreated cells (*: *p*  ≤  0.05; **: *p*  ≤  0.005; ns—non significant).

For this purpose, we used the bone explant culture model as it enabled us to investigate the effect of specific stimuli on bone cells in a system in which the cell–cell and cell–matrix interaction is intact. Using bone explant, we checked the effect of rIL-9 on the expression of MMP-1, -2, -3, -8, -9, -12, -13, and -16 (Figure 5A–F). We observed that IL-9 significantly enhanced the expression of collagenase (MMP1 and MMP13), gelatinase (MMP9), elastase (MMP12), and membrane-type metalloproteinases (MMP16). These observations suggest that IL-9 has the capacity to influence bone degradation by modulating the expression of MMPs in the bone cells.

## 3. Discussion

In RA, inflammatory cytokines including TNF-α, IL-6, IL-1, and IL-17 play a central role in modulating the process of bone remodeling by enhancing bone resorption and reducing bone formation [28]. Recently, elevated levels of IL-9 have been documented in arthritic conditions such as psoriatic arthritis (PsA) and RA [29,30]. IL-9 accentuates inflammation in PsA by activating γδ T cells and in RA, by promoting the function of Th1 and Th17 cells [21,31]. However, in RA, another report showed that IL-9-produced by type 2 innate lymphoid cells (ILC2s) contributes to induction of the resolution of inflammation [32]. Various cellular sources of IL-9 have been documented in literature [23]. Our group and others have shown the enrichment of IL-9-producing Th-9 cells in the peripheral blood as well as synovial fluid of patients with RA [20,21]. Rauber et al. also observed the expression of IL-9 in Lin+ cells in the synovial tissue of patients with active RA [32]. However, they observed a shift in the expression of IL-9 from Lin+ cells to Lin- ILC2 cells in the patients, who were in the clinical remission phase [32]. Thus, it appears from these observations that the cellular source of IL-9 dictates it role as protective or pathogenic.

In this study, we showed that IL-9 has the potential to enhance the RANKL-mediated osteoclastogenesis. IL-9 facilitates osteoclast differentiation in cells derived from HC as well as in patients with RA by enhancing the expression of osteoclast-specific genes. Here, we showed that IL-9 promotes osteoclast formation and bone resorptive capacity irrespective of its cellular source (PB or SF). Moreover, we showed that IL-9 augments osteoclast formation and function in RA by inducing the production of TNF-α, a potent osteoclastogenic cytokine.

Transcriptome analysis revealed that the IL-9 differentially modulates the expression of genes to facilitate osteoclast formation and function. IL-9 upregulates the expression of ephrinB2, an important molecule in mediating bone homeostasis and a target of NFATc1. It has been observed that during RANKL-mediated osteoclastogenesis, the expression of ephrinB2 gets upregulated [33]. In our study, we also found RANKL-induced upregulation of ephrinB2. In the bone remodeling cycle, the bidirectional interaction between ephrinB2 and its receptor EphB4 expressed on osteoblast cells has been considered to regulate the transition from the bone destruction phase to a bone formation phase. Another gene, which was upregulated by IL-9 stimulation along with MCSF and sRANKL is Krueppel-like factor 2 (KLF-2). Recent studies have shown the importance of KLF-2 in regulating autophagy [34]. The differentiation of osteoclasts from their precursor cells is a complex phenomenon and autophagy plays an important role in regulating this process. Autophagy is essential for the cell survival and for recycling of the organelles and proteins during osteoclastogenesis. Thus, IL-9 might influence autophagy by regulating the expression of KLF-2 during osteoclast differentiation process.

In addition to autophagy, the osteoclast differentiation and maturation process requires active metabolic reprogramming due to the synthesis of a variety of proteins involved in fusion, proton pumps, ion channels, and proteases, etc. Indeed, KEGG pathway analysis of transcriptomics data revealed that IL-9 induced differentially expressed genes which are mostly involved in the metabolic pathway. Recent studies have appreciated the importance of lipid metabolism in bone remodeling. Enhanced osteoclastogenesis has been observed in mice with high fat diets [35,36,37]. Additionally, high-fat-diet-fed animals showed severe destruction of joints compared to animals with normal diets [38]. It appears from our results that IL-9 has the potential to regulate lipid metabolism during osteoclast differentiation by upregulating the expression of *ABCA7* and *ACSM4,* which are involved in lipid metabolism [39,40,41,42].

Another important step in the formation of osteoclasts is the fusion of mononuclear cells. During osteoclastogenesis, involvement of cadherin-6 in cell–cell interaction and K+ channel in multinucleation has been observed [43,44]. Here, we showed that IL-9 influence the fusion process in osteoclast differentiation by regulating the expression of KCN4 and CDK6.

Extensive literature supports the importance of MMPs in bone and cartilage degradation. Inflammatory cytokines such as TNF-α and IL-1 have been known to modulate the expression of MMPs [45,46,47]. Here, we demonstrate for the first time that IL-9 also has the potential to regulate the expression of MMP 1, 9,12, 13, and 16 in bone cells. Together, our observations suggest that IL-9 may influence the bone degradation in RA by promoting osteoclastogenesis and by modulating the expression of MMPs.

In the clinical course of RA, progressive bone erosion results in functional disability. Disease-modifying antirheumatic drugs help in reducing the ongoing inflammation but fail to halt the bone destruction in RA [48]. Biologics targeting potent osteoclastogenic cytokine-like TNF-α have revolutionized the treatment of RA as it prevents bone erosion [49,50,51]. Based on our observation, we conclude that IL-9 could be a good therapeutic target for preventing progressive erosive arthritis in RA.

## 4. Materials and Methods

### 4.1. Study Subjects

The study was conducted with approval from Institute Ethics Committee (IEC-490/01.09.2017). After obtaining informed consent, subjects were recruited from the Department of Orthopedics OPD and Department of Rheumatology OPD of AIIMS, New Delhi. Diagnosis was made on the basis of ACR criteria 1987. All recruited RA patients had active disease. For this study, we collected synovial fluid (SF) and autologous peripheral blood (PB) from RA patients during RA flares. Specimens for cell culture were collected in heparinized tubes (BD, Franklin Lakes, NJ, USA). Fifteen age-matched healthy controls (HC) who were free from any acute or chronic ailment and were not on medication at the time of enrolment were recruited.

### 4.2. Cell Separation, Culture, and Stimulation

Synovial fluid mononuclear cells (SFMC) and peripheral blood mononuclear cells (PBMCs) were isolated using density gradient centrifugation with Lymphoprep (Axis-Shield, Oslo, Norway). Monocytes were enriched from mononuclear cells due to their ability to adhere. Briefly, cells were plated on tissue culture plates for 12 h followed by the removal of non-adherent cells. Adherent cells were grown in α-minimum essential media (MEM) (Gibco, Grand Island, NY, USA) containing 10% fetal bovine serum (FBS) (Gibco, Grand Island, NY, USA), 100 U/mL penicillin, and 100 µg/mL streptomycin (Sigma-Aldrich, St. Louis, MO, USA). Cells were kept in humidified 5% CO_2_ incubator at 37 °C. Cells were stimulated as indicated with soluble (s)-RANKL(ProSpec, Israel), M-CSF (R&D Systems, Minneapolis, MN, USA) and rIL-9 (ProSpec, Israel).

### 4.3. TRAP Staining

A total of 2 × 10^6^ cells was plated in 1 mL of complete medium in each well of 24-well plates. After 12 h, non-adherent cells were removed and adherent cells were kept in fresh complete medium. Adherent cells were then stimulated as described in figure legends with M-CSF alone or along with sRANKL in presence and absence of rIL-9 for 21 days. After 21 days, cell supernatant was removed and cells were fixed. Fixed cells were then stained using acid phosphatase, Leukocyte (TRAP) Kit (Sigma, St. Louis, MO, USA) as recommended by manufacturer’s protocol. Multinucleated (three or more nuclei) TRAP-positive cells were scored as osteoclasts.

### 4.4. Bone Explant Culture

Bone samples were obtained under sterile conditions from subjects undergoing orthopedic surgery for non-pathological fractures. Bone samples were washed several times with phosphate buffer saline (PBS) containing antibiotics. Samples were broken into small pieces. Bone explant cultures were performed in complete αMEM medium (Gibco, Grand Island, NY, USA) supplemented with 10% FBS (Gibco, Grand Island, NY, USA), 2 mM l-glutamine, 100 U/mL penicillin, and 100 µg/mL streptomycin (Sigma-Aldrich, St. Louis, MO, USA). Each piece of bone was kept in one well of a 6-well plate and in the presence or absence of 100 ng/mL IL-9 for 5 days. Bone explant cultures were maintained in humidified 5% CO_2_ incubator at 37 °C.

### 4.5. Real Time PCR

A total of 2 × 10^6^ cells derived from peripheral blood or synovial fluid were seeded per well of a 6-well culture plate and then stimulated with either MCSF or MCSF along with sRANKL in the presence or absence of rIL-9. RNA was extracted using GeneJET RNA Purification Kit (Thermo Fisher Scientific, Waltham, MA, USA), according to the manufacturer’s protocol. cDNA was prepared from equal quantity of RNA by using Revert Aid First strand cDNA synthesis kit (Thermo Fisher Scientific, Waltham, MA, USA). Quantitative RT-PCR was performed using Powerup Sybr Master Mix (Applied Biosystem, Waltham, MA, USA) with the primers listed in Table 2. RT-PCR was performed using the QuantStudio 5 Real-Time PCR Systems (Applied Biosystems, Waltham, MA, USA). For amplification of all the genes, an initial enzyme activation step of 2 min at 50 °C and denaturation step of 5 min at 95 °C was common. However, the annealing cycles for each gene were different. For *EFNB2* and *ACSM4* (40 cycles at 95 °C for 15 s and at 63 °C for 1 min); *KCNA3*, *CDH6*, *KLF2, ACSM4* (40 cycles at 95 °C for 15 s and at 67 °C for 1 min); *NFATc1*, *CTSK*, *ACP5*, *GAPDH* (40 cycles at 95 °C for 15 s and at 56 °C for 1 min); *MMP1*, *MMP2*, *MMP3*, *MMP8*, *MMP9*, *MMP12*, (40 cycles at 95 °C for 15 s, 58 °C for 1 min); and *MMP13* and *MMP16* (40 cycles at 95 °C for 15 s, 56 °C for 1 min). GAPDH was used as a normalization control. The fold change was calculated using 2^−ΔΔ*CT*^.

### 4.6. Cytokine ELISA

A total of 2 × 10^6^ cells derived from PB or SF were seeded per well of a 6-well culture plate and then stimulated with either M-CSF or M-CSF along with sRANKL in the presence or absence of rIL-9 for 4 days. Supernatants of stimulated cells were harvested and analyzed for TNF-α, IL-6, and IL-1β using ELISA Kit (Invitrogen Human TNF-α/IL-6/IL-1β uncoated ELISA kit; Invitrogen, Waltham, MA, USA) as per the manufacturer’s protocol.

### 4.7. Bone Resorption Assay

1 × 10^6^ cells derived from PB or SF were seeded per well of a 24-well fluoresceinated calcium-phosphate-coated plate (Cosmo Bio Co., LTD, Tokyo, Japan). Cells were cultured in phenol-red-free DMEM medium (Gibco, Grand Island, NY, USA) containing 10% FBS (Gibco, Grand Island, NY, USA) as described by manufacturer’s protocol. For differentiation, cells were stimulated as indicated for 24 days. On every fourth day, half of the medium was replaced with fresh medium containing M-CSF, sRANKL, or r-IL-9. Evaluation of bone resorption activity was done by measuring the fluorescence intensity of the conditioned medium at an excitation wavelength of 485 nm and emission wavelength of 590 nm as per the instructions of the manufacturer’s protocol using a Tecan plate reader.

### 4.8. RNA Sequencing and Analysis

A total of 2 × 10^6^ cells were seeded per well of a 6-well culture plate and then stimulated as indicated. Total RNA was isolated using TRIzol (Thermo Fisher Scientific, Waltham, MA, USA). The quality of RNA was checked on the Agilent TapeStation system using the manufacturer’s protocol. The first strand of cDNA was synthesized followed by preparation of mRNA library. Sequencing was performed using Illumina HiSeqX10. FastQc (Version 0.11.5) was used for checking the quality of the reads including distribution of base quality score, distribution of sequence quality score, average base content per read, and distribution of GC in the reads (Table 3). The mapping of reads to the human reference genome was done using Minimap2 software. Samtools was used for quantifying the level of gene expression. Differential expression analysis was performed using DEseq software. Expression plots were made using R scripts.

### 4.9. Statistical Analysis

Data are presented as means ± SD. Comparison between the groups was performed by employing Student’s *t*-test. *p* values ≤ 0.05 were considered significant. Statistical significance of the results was determined with help of the Graph Pad Prism 5 software (GraphPad Software Inc., La Jolla, CA, USA).

## 5. Conclusions

Our study strongly suggest that IL-9 has the potential to mediate structural damage in inflammatory bone diseases such as RA by the enhancing the differentiation of osteoclasts, its bone resorptive capacity, and by modulating the expression of MMPs. Thus, blocking or inhibiting the IL 9 pathway might be an attractive immunotherapeutic strategy for RA in preventing bone loss.

## Figures and Tables

**Table 1:** Characteristics of rheumatoid arthritis patients included in the study.

Patient	Age/Sex	Duration of Disease (Years)	DAS28 ESR	ESR (mm/1st Hour)	CRP(mg/lit)	Treatment	Joint Erosions
1	43/F	9.4	6.1	56	43.6	Mtx	YES
2	54/F	16.7	5.9	34	11.9	Mtx, Inf	YES
3	31/M	4.2	4.5	22	13.4	Mtx	NO
4	45/F	8.4	4.2	59	39.1	Inf	YES
5	49/F	5.9	4.6	31	24.9	Ada, Mtx	NO
6	56/F	11.5	6.2	42	28.2	Mtx	YES
7	36/M	6.5	6.5	47	33.9	Mtx	YES
8	41/F	8.4	5.1	41	27.3	Lfu	YES
9	47/M	10.	5.2	11	1.3	Eta	NO
10	29/M	8.5	4.2	21	4.3	Mtx, Inf	YES
11	39/M	4.9	5.6	29	5.6	Mtx	YES
12	46/F	9.8	6.3	27	3.8	Mtx, Inf	YES
13	54/F	13.2	5.7	17	0.4	Mtx, Inf	YES
14	48/F	11.5	4.1	15	1.9	Mtx, Inf	YES
15	58/F	17.5	4.0	24	2.5	Mtx, Inf	YES

DAS28, disease activity score 28—joint assessment; CRP, C-reactive protein; ESR, erythrocyte sedimentation rate; Mtx, methotrexate; Inf, infliximab; Eta, etanercept; Lfu, leflunomide.

**Table 2:** List of primers and their sequences.

Gene	Primer Sequence
EFNB2	Forward primer: 5′ TCCAACAAGACGTCCAGAAC 3′ Reverse primer: 5′ CGTCTGTGCTAGAACCTGGAT 3′
KCNA3	Forward primer: 5′ TTGGTCTGCCTATGCCCTTG 3′ Reverse primer: 5′ CAGCCCACTTGCAAAACAGG 3′
CDH6	Forward primer: 5′ TTTCGTTTTCCTTGGCCCCT 3′ Reverse primer: 5′ CGCCGTGTTGTCTTTGTTGT 3′
ABCA7	Forward primer: 5′ TGAGGTCAGATACGGAGGCT 3′ Reverse primer: 5′ TTTTCAGGACACGGTCGAGG 3′
KLF2	Forward primer: 5′ GTGGGCATTTTTGGGCTACC 3′ Reverse primer: 5′ CCCAGTTCCAAGCAACCAGA 3′
ACSM4	Forward primer: 5′ ACTGCTGCCACGATAAGAGG 3′ Reverse primer: 5′ GCCCAATACGGTACCCAGAG 3′
MMP1	Forward primer: 5′ AGCCATCACTTACCTTGCACT 3′ Reverse primer: 5′ CTGGGAAGCTGTGAGACACC 3′
MMP2	Forward primer: 5′ ATCCAGACTTCCTCAGGCGG 3′ Reverse primer: 5′ CCATTAGCGCCTCCATCGTAG 3′
MMP3	Forward primer: 5′ TGAAATTGGCCACTCCCTGG 3′ Reverse primer: 5′GGAACCGAGTCAGGTCTGTG 3′
MMP8	Forward primer: 5′CTCCCTGAAGACGCTTCCAT 3′ Reverse primer: 5′TCCAGGTAGTCCTGAACAGT 3′
MMP9	Forward primer: 5′ GTACTCGACCTGTACCAGCG 3′ Reverse primer: 5′ AGAAGCCCCACTTCTTGTCG 3′
MMP12	Forward primer: 5′ AACCAACGCTTGCCAAATCC 3′ Reverse primer: 5′ TTTCCCACGGTAGTGACAGC 3′
MMP13	Forward primer: 5′ TTGTTGCTGCGCATGAGTTC 3′ Reverse primer: 5′AAGTGGCTTTTGCCGGTGTA 3′
MMP16	Forward primer: 5′CTACTGCTGACCCTGTCTTCG 3′ Reverse primer: 5′ GCTTCAATGGATGGACGAGC 3′
NFATc1	Forward primer: 5′ CCGTAGGCTTGTTCATAG 3′ Reverse primer: 5′ GTAACCTATTGATGTGCTATTC 3′
CTSK	Forward primer: 5′ CAAATCCATCCTGCTCTTC 3′ Reverse primer: 5′ TATCACCACATCTGCTTCA 3′
ACP5	Forward primer: 5′ CTTCCACTATGGGACTGA 3′ Reverse primer: 5′ CCTCGATGTAAGTGACAG3′
GAPDH	Forward primer: 5′ AGTCAGCCGCATCTTCTTTT 3′ Reverse primer: 5′ CCCAATACGACCAAATCCGT 3′

**Table 3:** The number of clean reads in the MCSF + RANKL- and MCSF + RANKL + IL-9-treated group.

Sample	Number of Reads
MCSF_RANKL1_set1_R1	31,668,998
MCSF_RANKL1_set1_R2	31,668,998
MCSF_RANKL2_set2_R1	34,530,684
MCSF_RANKL2_set2_R2	34,530,684
MCSF_RANKL_IL9_set1_R1	30,689,185
MCSF_RANKL_IL9_set1_R2	30,689,185
MCSF_RANKL_IL9_set2_R1	39,599,229
MCSF_RANKL_IL9_set2_R2	39,599,229

## Data Availability

All data generated or analyzed during this study are included in this manuscript [and its Supplementary Information files].

## Supplementary Materials

The following are available online at https://www.mdpi.com/article/10.3390/ijms221910397/s1.
