# Supplementary material for: Interleukin-9 Facilitates Osteoclastogenesis in Rheumatoid Arthritis
Source: Int J Mol Sci. 2021 Sep 27;22(19):10397. doi: 10.3390/ijms221910397 (PMC8508938; doi:10.3390/ijms221910397)
Supplement: Supplementary file 1 [file ijms-22-10397-s001.zip › ijms-1345262-supplementary.pdf]

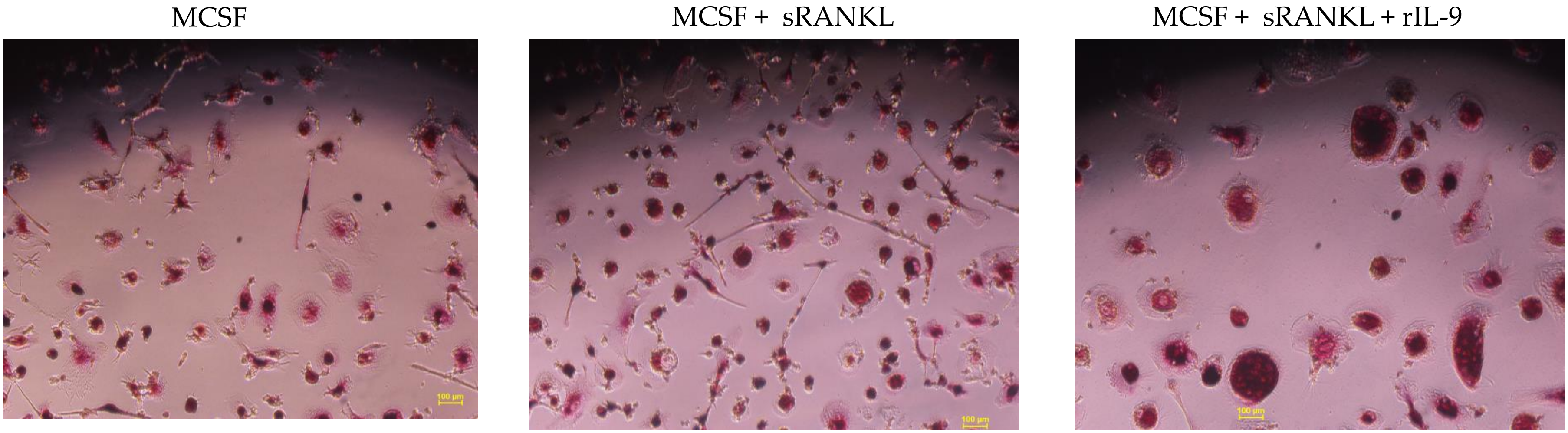

Supplementary Figure 1: Interleukin (IL)-9 enhances osteoclast formation in RA. Cells derived from peripheral blood (PB) of patients with RA were treated as indicated with macrophage colony-stimulating factor (M-CSF; 25 ng/mL) or soluble receptor activator of nuclear factor κB ligand (sRANKL; 50 ng/mL), or IL-9 (100 ng/mL) for 21 days. Cells were then fixed and stained for tartrate-resistant acid phosphatase (TRAP). Representative picture of multinucleated (≥3 nuclei) TRAP+ cells.

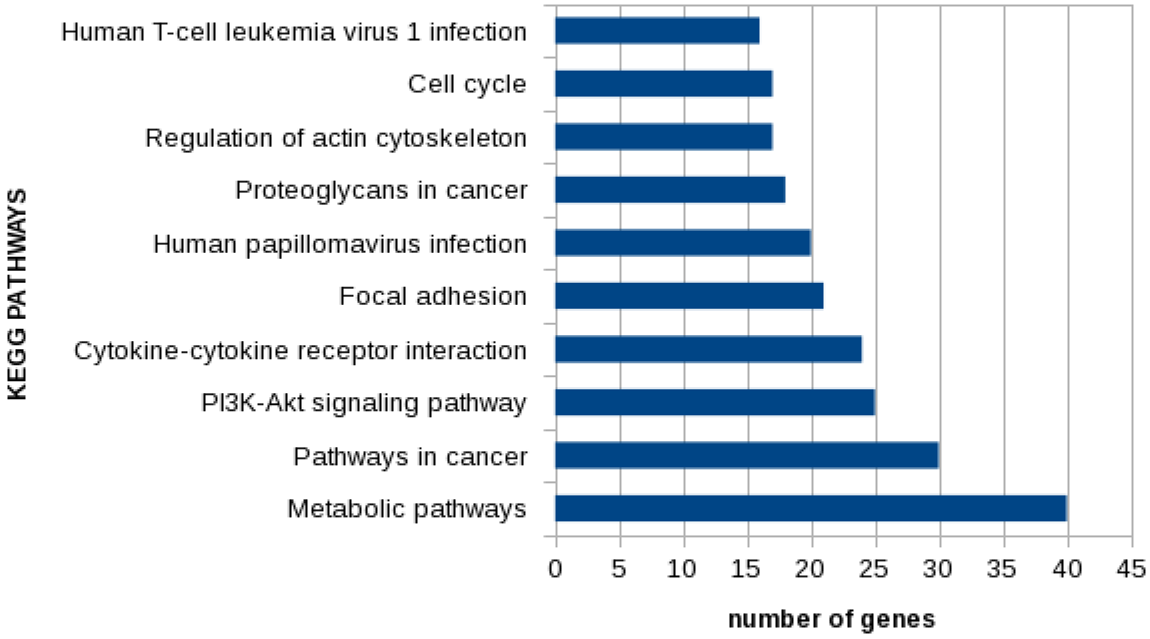

Supplementary Figure 2: KEGG pathway analysis of differentially expressed genes in cells treated with M-CSF and sRANKL in presence and absence of IL-9.
